# Supplementary material for: On the causes of gene-body methylation variation in Arabidopsis thaliana
Source: PLoS Genet. 2023 May 4;19(5):e1010728. doi: 10.1371/journal.pgen.1010728 (PMC10187938; doi:10.1371/journal.pgen.1010728)
Supplement: S1 Table — (PDF) [file pgen.1010728.s001.pdf]

S1 Table. Individuals sequenced.

Genotype	16C	4C	Total
Parent N	7	16	23
Parent S	9	16	25
F2 (NN × SS)	134	174	308
F2 (SS × NN)	132	174	306
