# Supplementary material for: On the causes of gene-body methylation variation in Arabidopsis thaliana
Source: PLoS Genet. 2023 May 4;19(5):e1010728. doi: 10.1371/journal.pgen.1010728 (PMC10187938; doi:10.1371/journal.pgen.1010728)
Supplement: S2 Table — (PDF) [file pgen.1010728.s002.pdf]

S2 Table. Average somatic deviations in F2 individuals.

	Gains	Losses
16C	0.00092	0.0744
4C	0.001	0.0724
Mean	0.00097	0.073
