# Supplementary material for: On the causes of gene-body methylation variation in Arabidopsis thaliana
Source: PLoS Genet. 2023 May 4;19(5):e1010728. doi: 10.1371/journal.pgen.1010728 (PMC10187938; doi:10.1371/journal.pgen.1010728)
Supplement: S3 Table — (PDF) [file pgen.1010728.s003.pdf]

S3 Table. Deviations in NN and SS backgrounds, separately for sites that are identical vs differ between N and S.

	Gains		Losses	
	Identical	Differ	Identical	Differ
NN	0.0011	0.0152	0.0738	0.1428
SS	0.00085	0.01470	0.0753	0.1558
