# Supplementary material for: On the causes of gene-body methylation variation in Arabidopsis thaliana
Source: PLoS Genet. 2023 May 4;19(5):e1010728. doi: 10.1371/journal.pgen.1010728 (PMC10187938; doi:10.1371/journal.pgen.1010728)
Supplement: S4 Table — (PDF) [file pgen.1010728.s004.pdf]

S4 Table. Linkage mapping results for deviations in NN $\times$SS cross.

	Top SNP	95% CI in Mb	Candidate genes (position)
Gains	Chr1:5038757	Chr1:4.0-5.5	RDR1 (5.1), SHH1 (5.2), IDNL1 (5.4), SUVH7 (6.1)
	Chr5:15932197	Chr5:15.8-17.6	VIM3 (15.8), AGO10 (17.6), SUVR2 (17.7)
Losses	Chr1:21740818	Chr1:8.0-23.6	VIM1 (21.4), NRPD1 (23.3)
	Chr4:5929511	Chr4:5.1-6.3	RDR2 (6.8), SUVH9 (7.8), MET2 (8.1)
	Chr5:16445720	Chr5:16.1-17.0	VIM3 (15.8), AGO10 (17.6), SUVR2 (17.7)
