# Supplementary material for: On the causes of gene-body methylation variation in Arabidopsis thaliana
Source: PLoS Genet. 2023 May 4;19(5):e1010728. doi: 10.1371/journal.pgen.1010728 (PMC10187938; doi:10.1371/journal.pgen.1010728)
Supplement: S5 Table — (PDF) [file pgen.1010728.s005.pdf]

S5 Table. Epimutation rates using data from S14 Fig.

	Gains (%)		Losses (%)	
Line	NN $\times$ SS	SS $\times$ NN	NN $\times$ SS	SS $\times$ NN
N	0.03	0.04	0.30	0.11
S	0.06	0.03	0.27	0.23
