# Supplementary material for: On the causes of gene-body methylation variation in Arabidopsis thaliana
Source: PLoS Genet. 2023 May 4;19(5):e1010728. doi: 10.1371/journal.pgen.1010728 (PMC10187938; doi:10.1371/journal.pgen.1010728)
Supplement: S6 Table — (PDF) [file pgen.1010728.s006.pdf]

S6 Table. Epimutation rates using data from S16 Fig.

	Gains (%)		Losses (%)	
Line	NN × SS	SS × NN	NN × SS	SS × NN
N	0.55	0.38	0.71	0.20
S	0.40	0.27	0.68	0.61
