# Supplementary figures and images for: On the causes of gene-body methylation variation in Arabidopsis thaliana
Source: PLoS Genet. 2023 May 4;19(5):e1010728. doi: 10.1371/journal.pgen.1010728 (PMC10187938; doi:10.1371/journal.pgen.1010728)

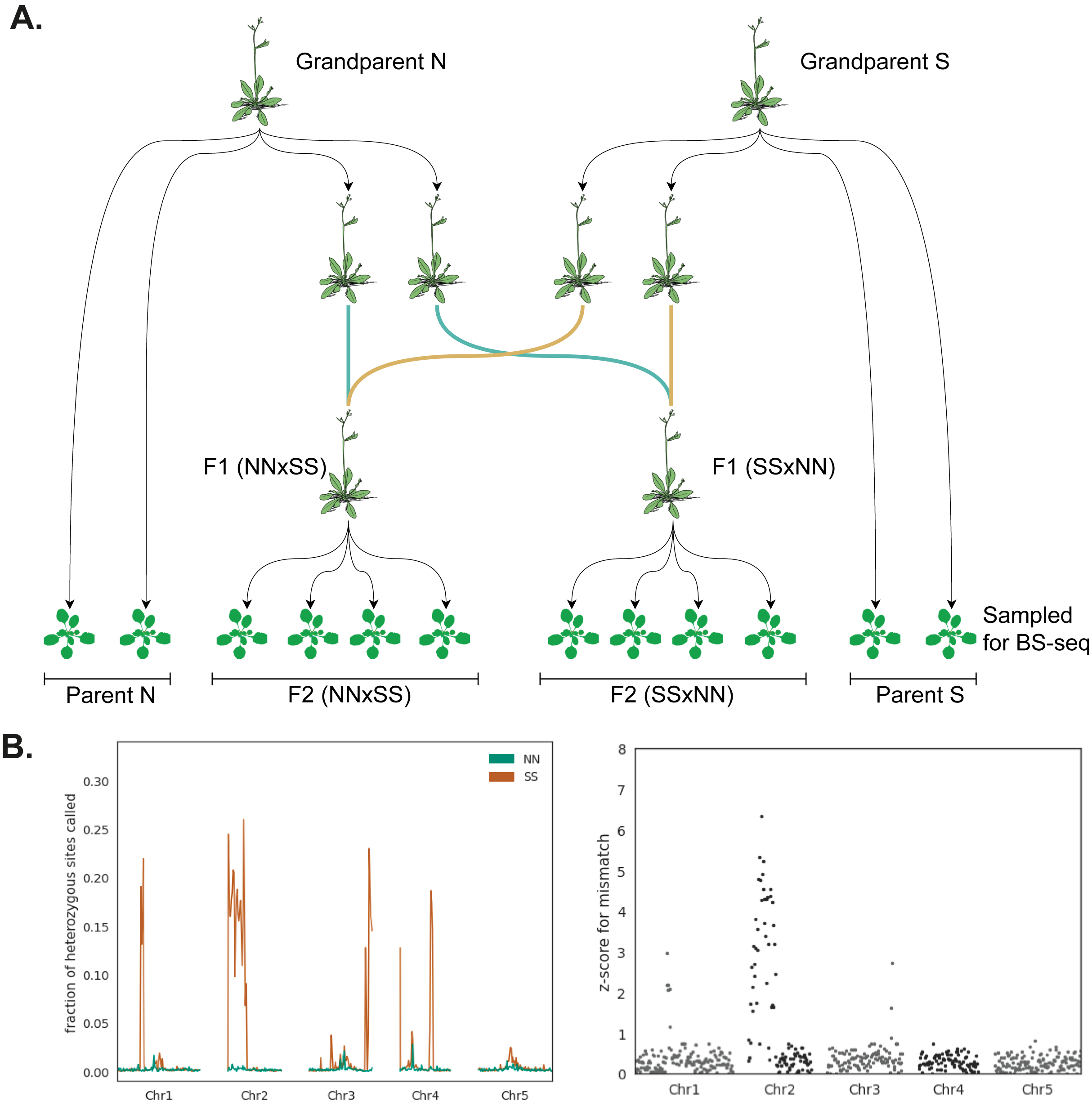

Supplement: S1 Fig — (A) Reciprocal F2 design. (B) The left panel shows evidence for residual heterozygosity in the parental lines in the 1001 Genomes data. The right panel shows region where different SNPs are segregating in the reciprocal F2 populations. (TIF) [file pgen.1010728.s007.tif]

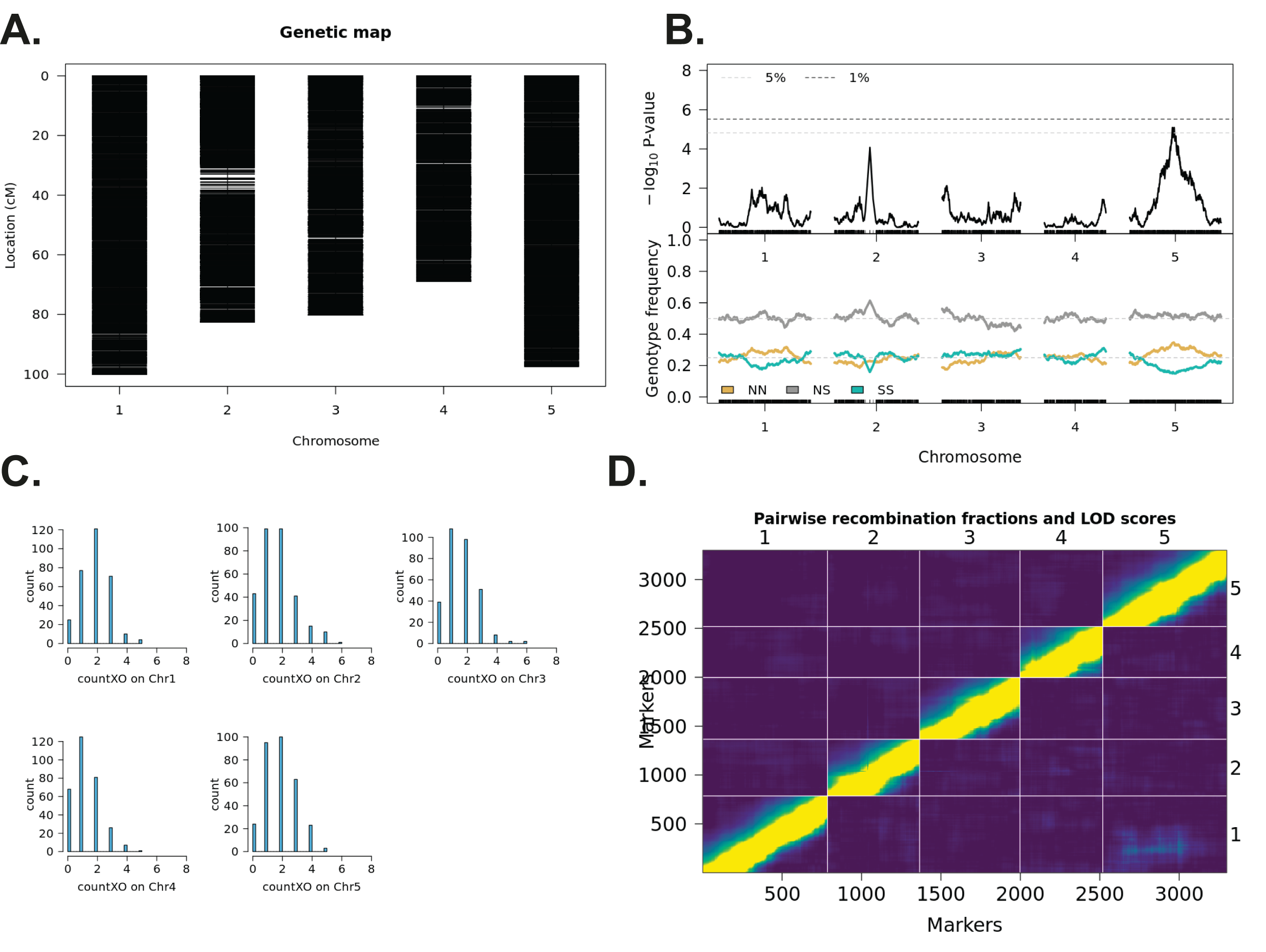

Supplement: S2 Fig — (A) Genetic map and markers. (B) Segregation distortion in the cross and genotype frequencies across the chromosome. (C) Number of crossovers per chromosome in the genetic map. (D) Pairwise recombination fraction (upper left triangle) and LOD scores for the markers. (TIF) [file pgen.1010728.s008.tif]

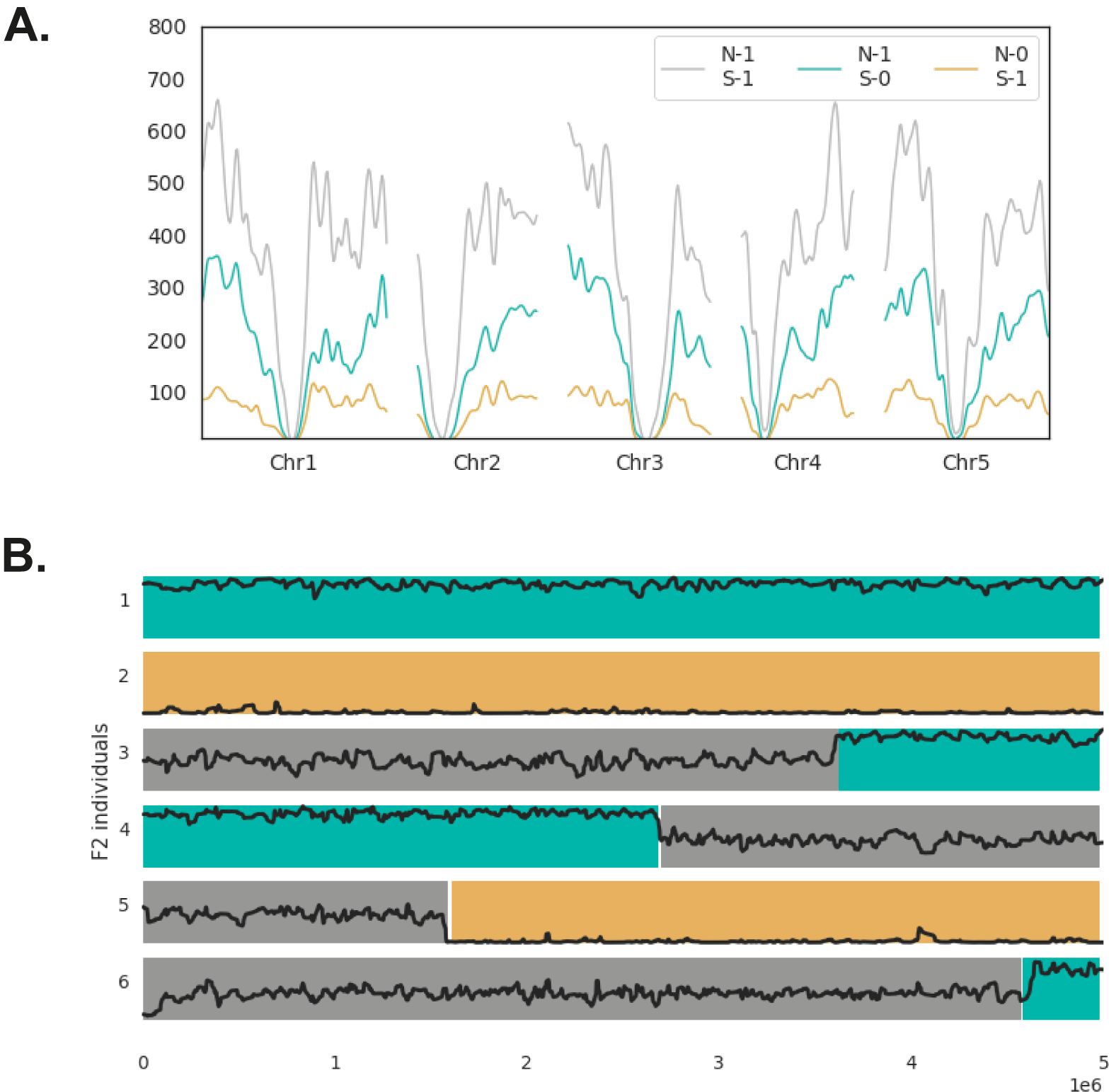

Supplement: S3 Fig — (A) Distribution of methylated CG sites the genome in 200 kb windows, separately for 213178 sites methylated in both parents (N-1 S-1), 109868 sites methylated only in the northern parent (N-1 S-0), and 39682 sites methylated only in the southern parent (N-0 S-1). (B) Genotype and relative methylation levels for 6 F2 individuals along chromosome 1. Genotypes are given by colors (NN is turquoise; SS is yellow; NS is grey), relative methylation levels by black curve. (TIF) [file pgen.1010728.s009.tif]

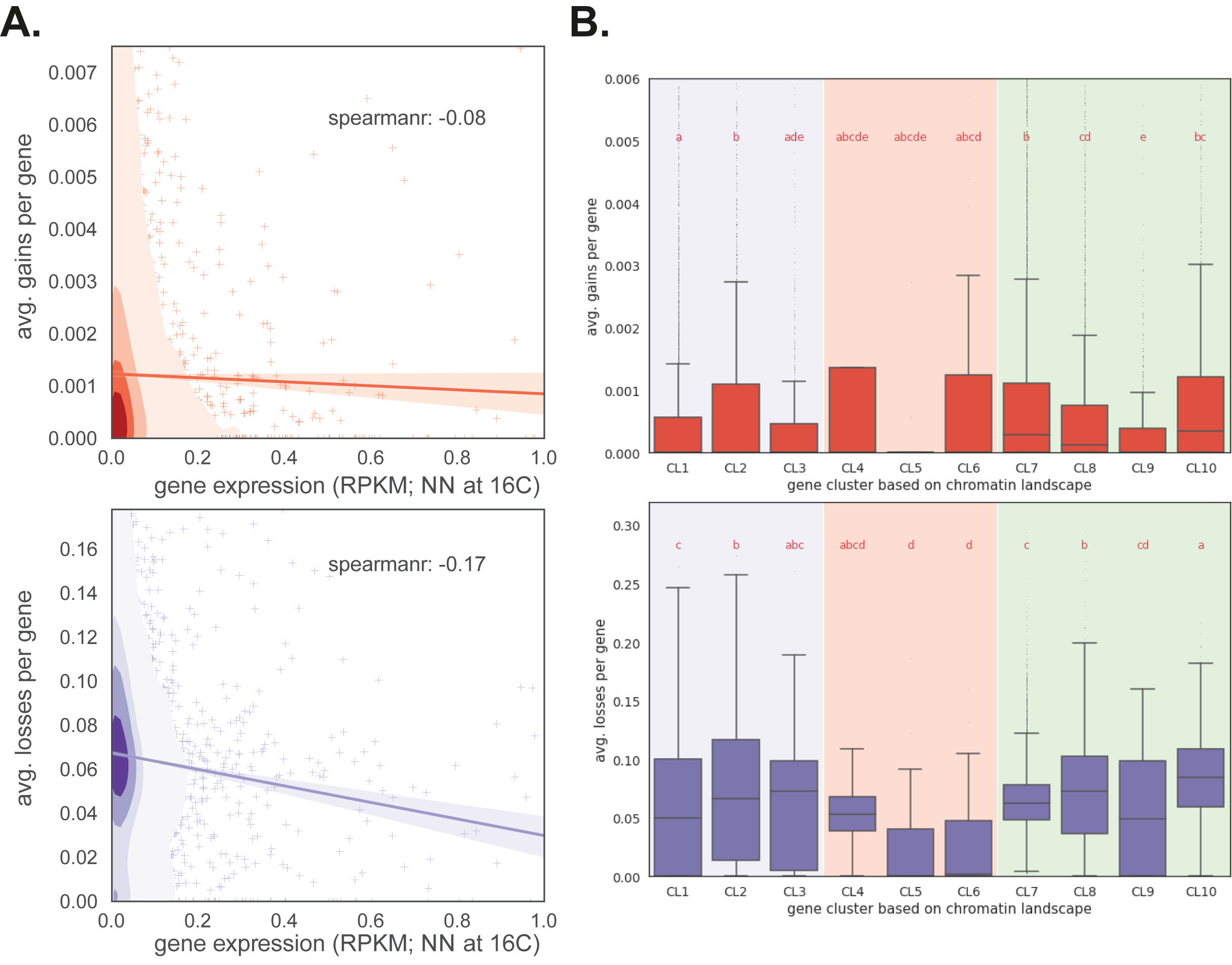

Supplement: S4 Fig — Average gains (top-panel) and losses (bottom-panel) for each gene (in NN background) plotted as a function of: (A) gene expression for NN genotype at 16°C (data from reference [3]); (B) ten annotated chromatin states (cf. Table 2 in reference [55]). Clusters CL1–CL3 represent genes in facultative heterochromatin and with Polycomb-like silencing. Clusters CL4–CL6 are genes with heterochromatic marks. Clusters CL7–CL10 are expressed genes. (TIF) [file pgen.1010728.s010.tif]

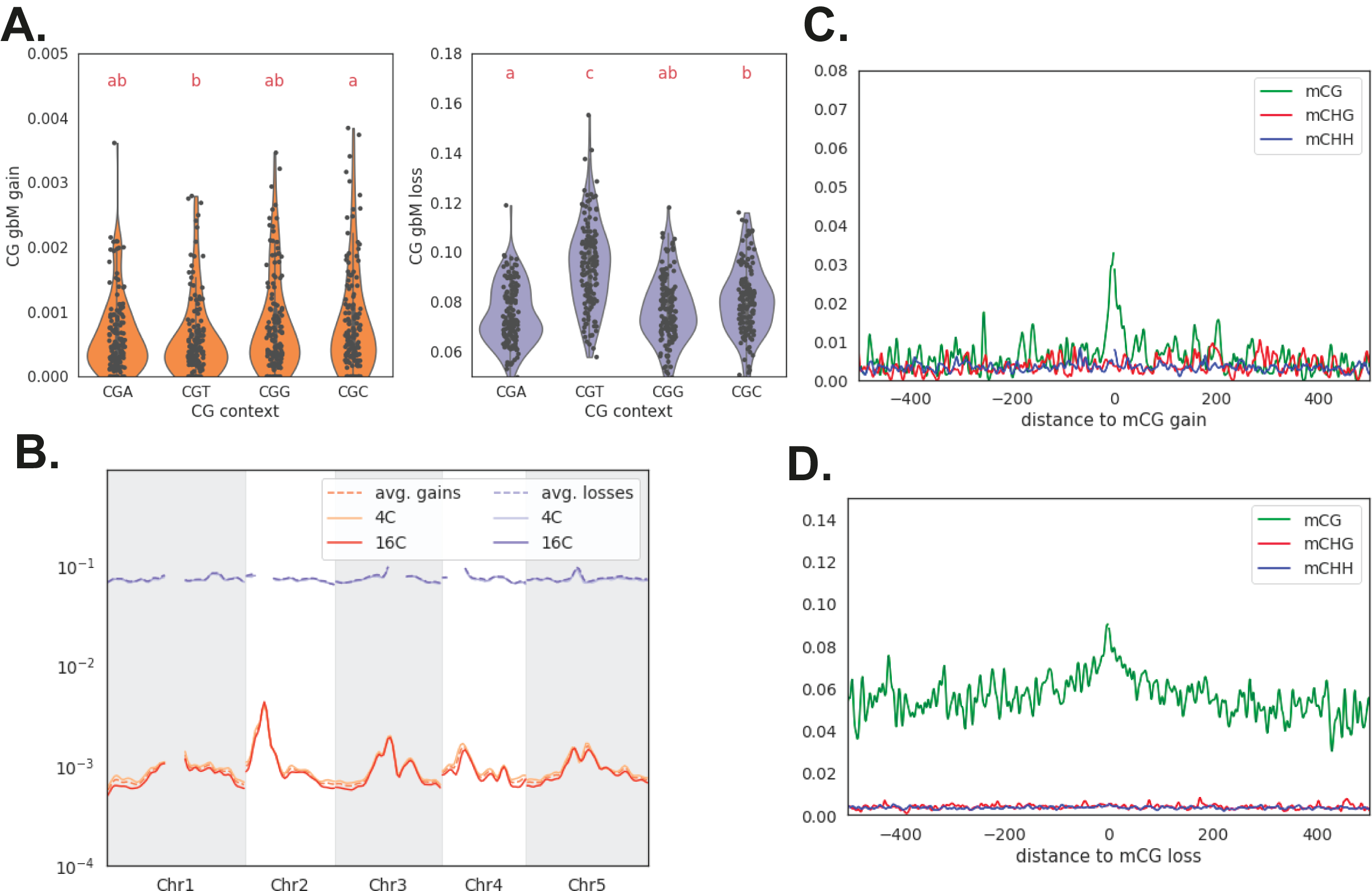

Supplement: S5 Fig — (A) Gains and losses separated by four contexts (CGA, CGT, CGG and CGC). (B) Average gains and losses across the genome at different temperatures. (C) Methylation levels at (gain at previously unmethylated) CG, CHG and CHH sites near a mCG gain site. (D) Methylation levels at CG (loss at previously methylated), CHG and CHH sites near a CG loss site. (TIF) [file pgen.1010728.s011.tif]

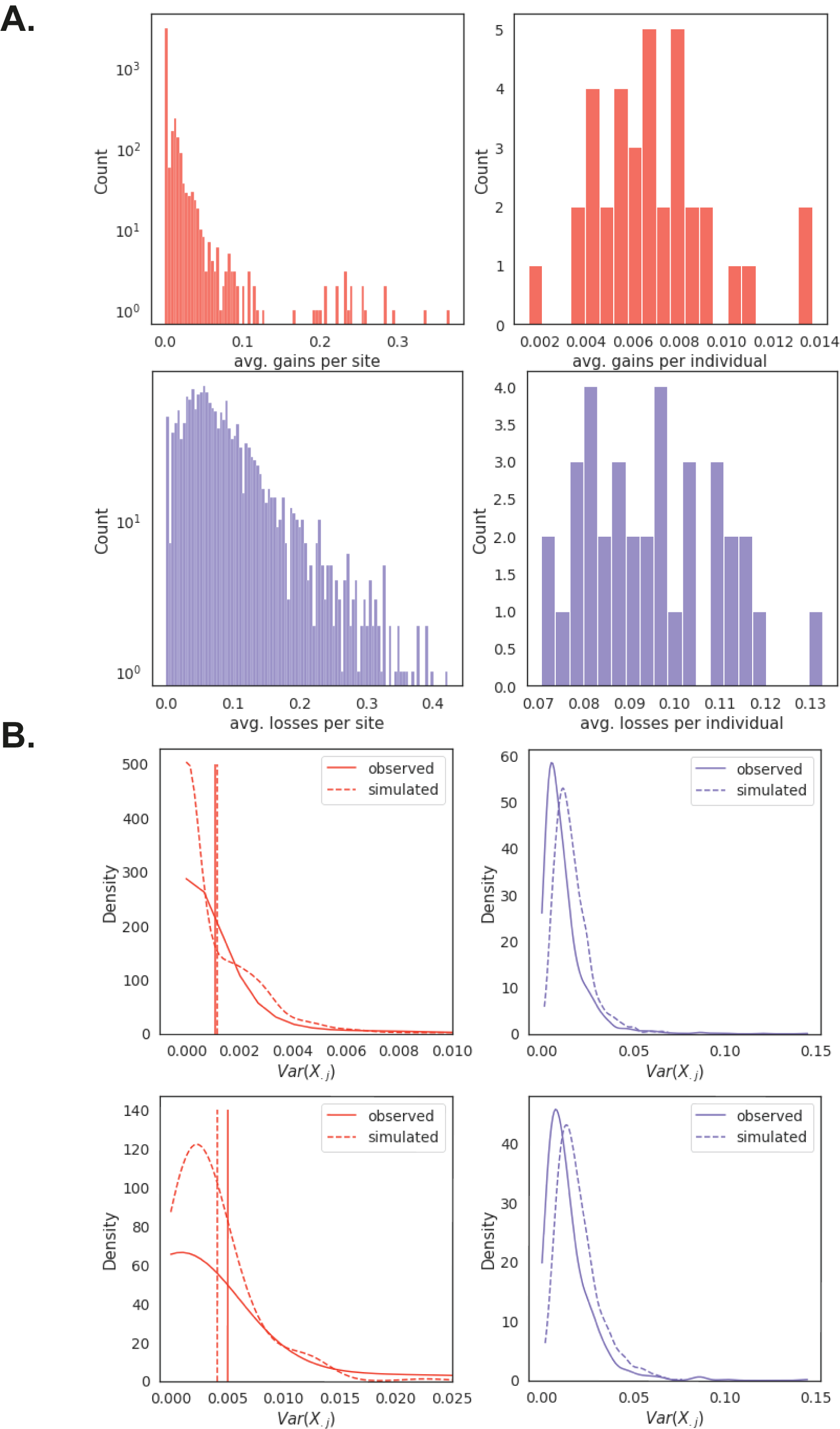

Supplement: S6 Fig — (A) Distribution of average deviations per site and per individual using data from chromosome 1 NN genotypes as an example. (B) Distribution of the variance between individuals across sites, Var(X.j), in data and in simulations. Top row shows the distribution for all sites, bottom row only for sites that are differentially methylated between N and S. (TIF) [file pgen.1010728.s012.tif]

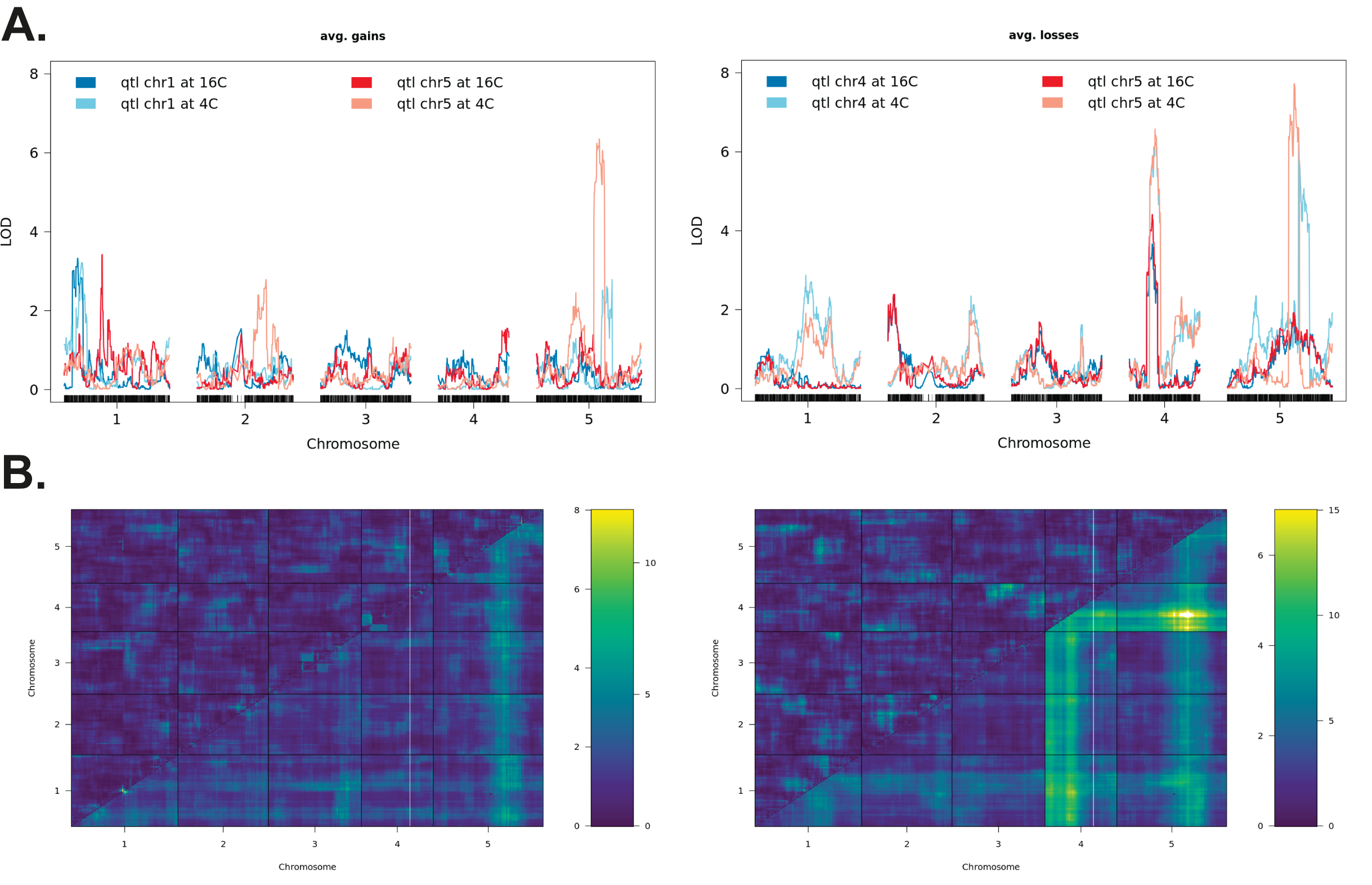

Supplement: S7 Fig — (A) Composite Interval Mapping was applied to four different gain phenotypes and four different loss phenotypes in order to refine peaks. For each of the four major QTL identified by combining results across 500 kb windows (two for gains and two for losses, see Fig 4) deviations were averaged over regions showing QTL effect at two temperatures. (B) Testing for epistasis on the QTLs for somatic deviations (using the “scantwo” function in R/qtl). Two QTLs on Chr1 and Chr5 for gains and three QTLs on Chr1, Chr4 and Chr5 for losses. The bottom triangle is the LOD scores for the full model including the interaction effect, upper triangle is LOD scores for only the interaction. (TIF) [file pgen.1010728.s013.tif]

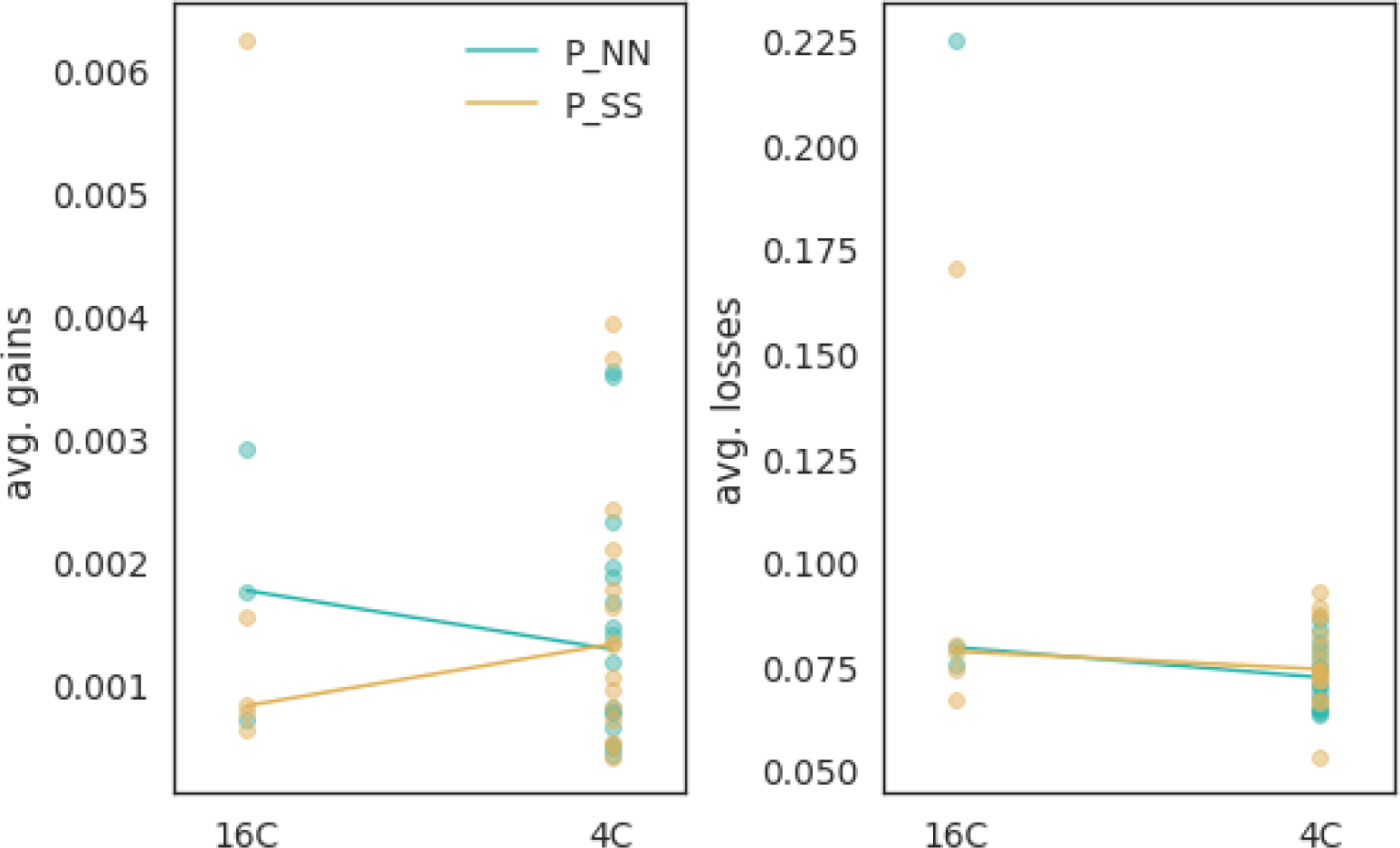

Supplement: S8 Fig — Reaction norms for average gains and losses for parental strains. (TIF) [file pgen.1010728.s014.tif]

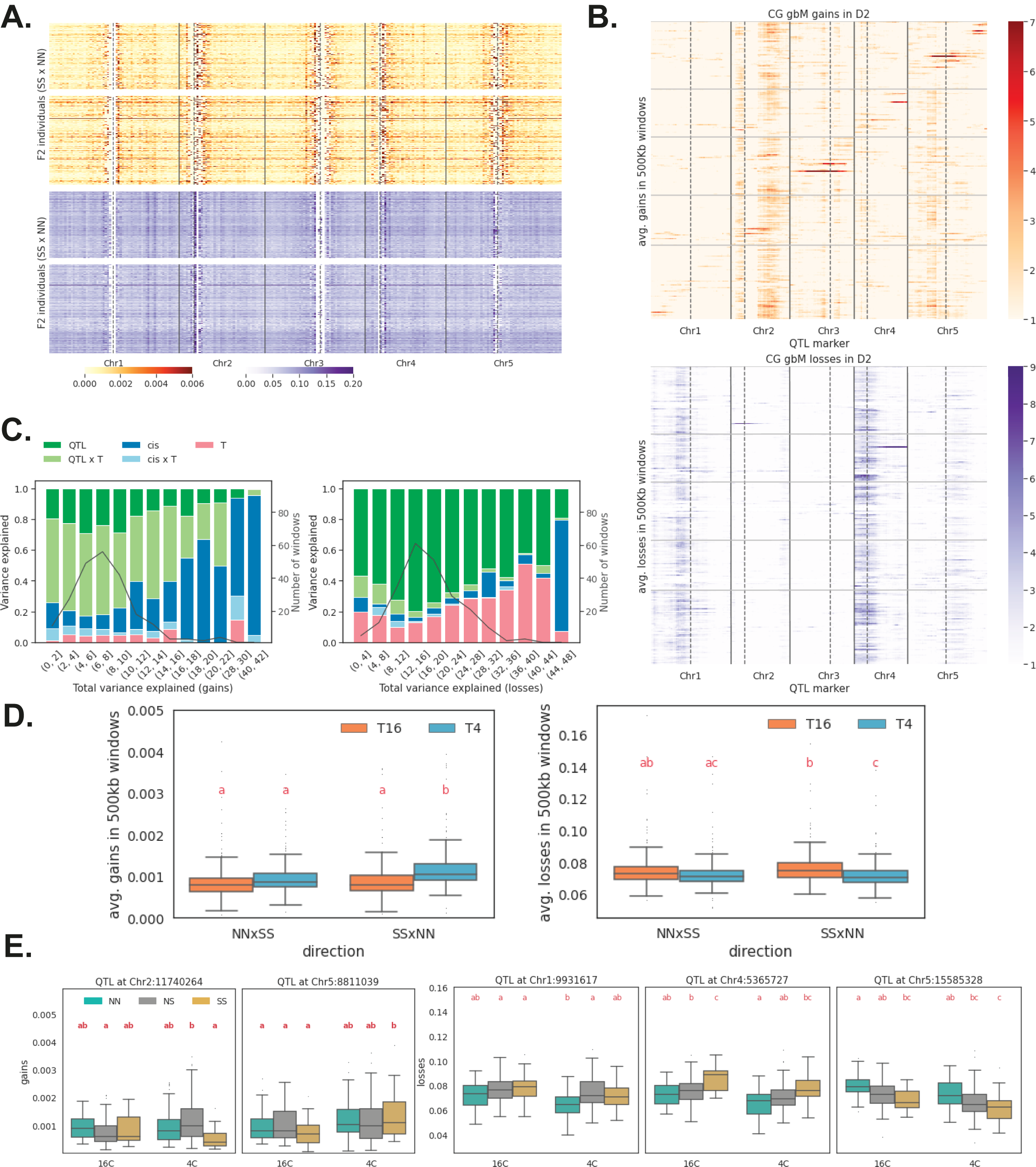

Supplement: S9 Fig — (A) Average deviations in 500 kb windows across genome (cf. Fig 3). (B) QTL mapping for gains and losses (cf. Fig 4). (C) Variance-partitioning results (cf. Fig 4). (D) Temperature effects on average gains and losses (in NN background) for both directions. (E) Genotypic effects for two gain QTL and three loss QTLs (cf. Fig 5). (TIF) [file pgen.1010728.s015.tif]

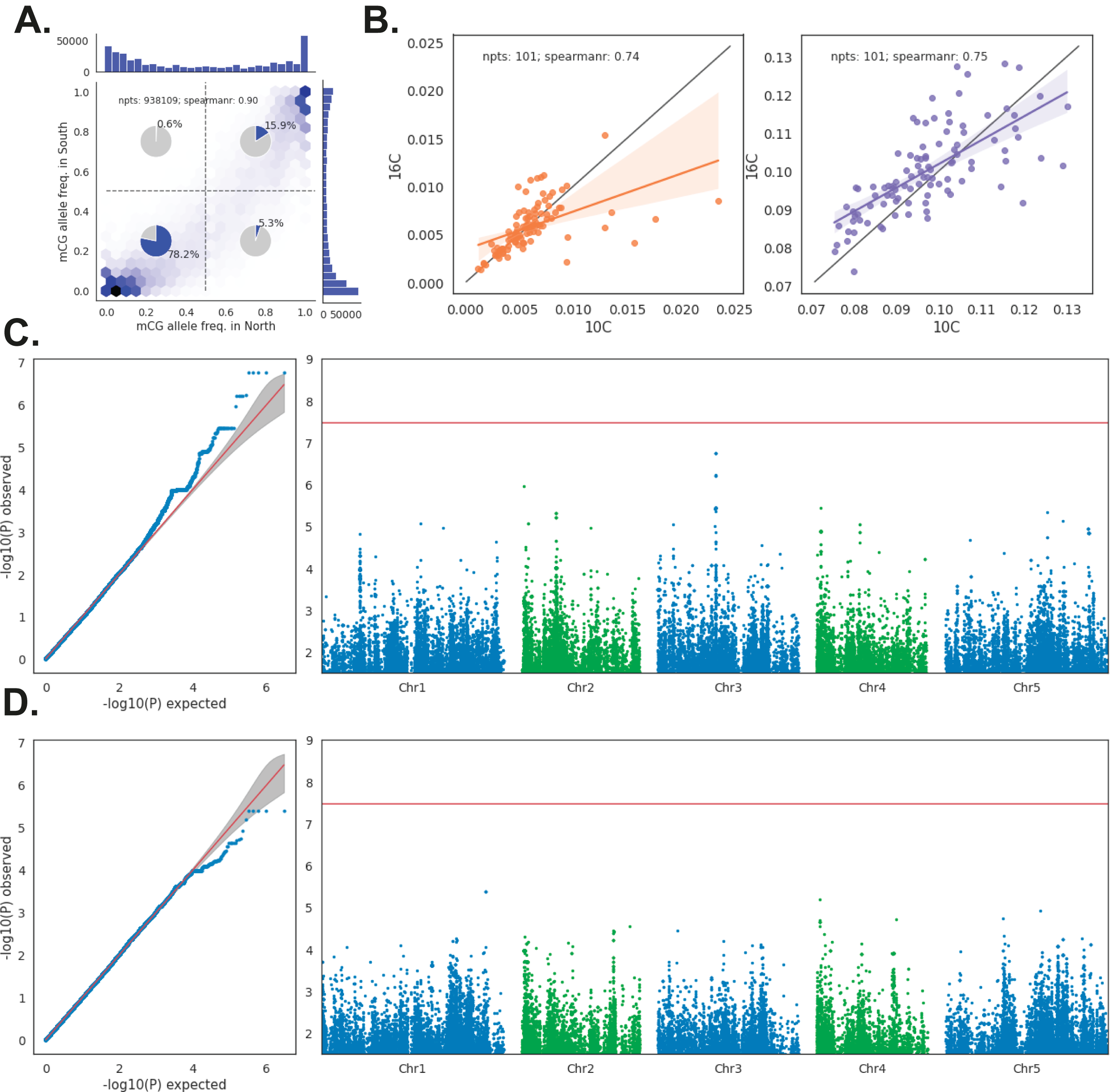

Supplement: S10 Fig — (A) mCG allele frequencies in populations from northern and southern Sweden [3]. (B) Correlation between genome-wide deviations between 10°C and 16°C. (C) GWAS for genome-wide gains. (D) GWAS for genome-wide losses. (TIF) [file pgen.1010728.s016.tif]

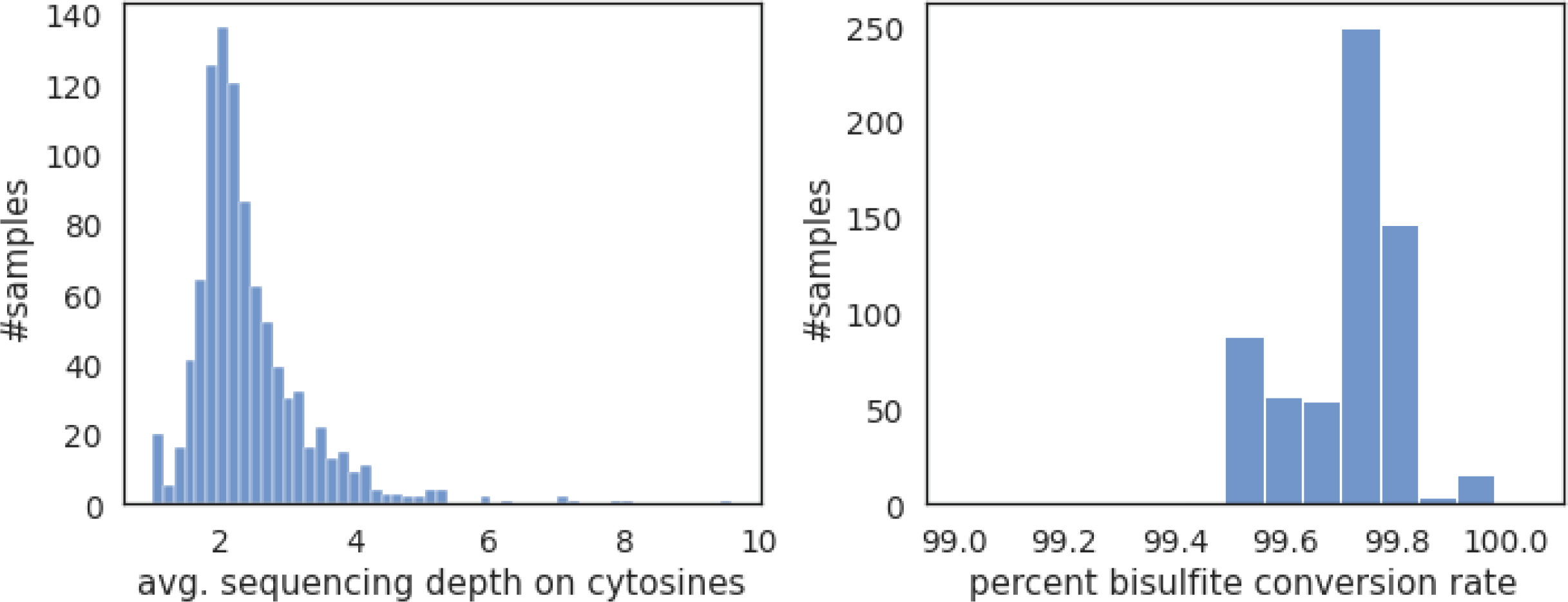

Supplement: S11 Fig — Average sequencing depth and bisulfite conversion estimated through chloroplast genome across 600 F2 individuals. (TIF) [file pgen.1010728.s017.tif]

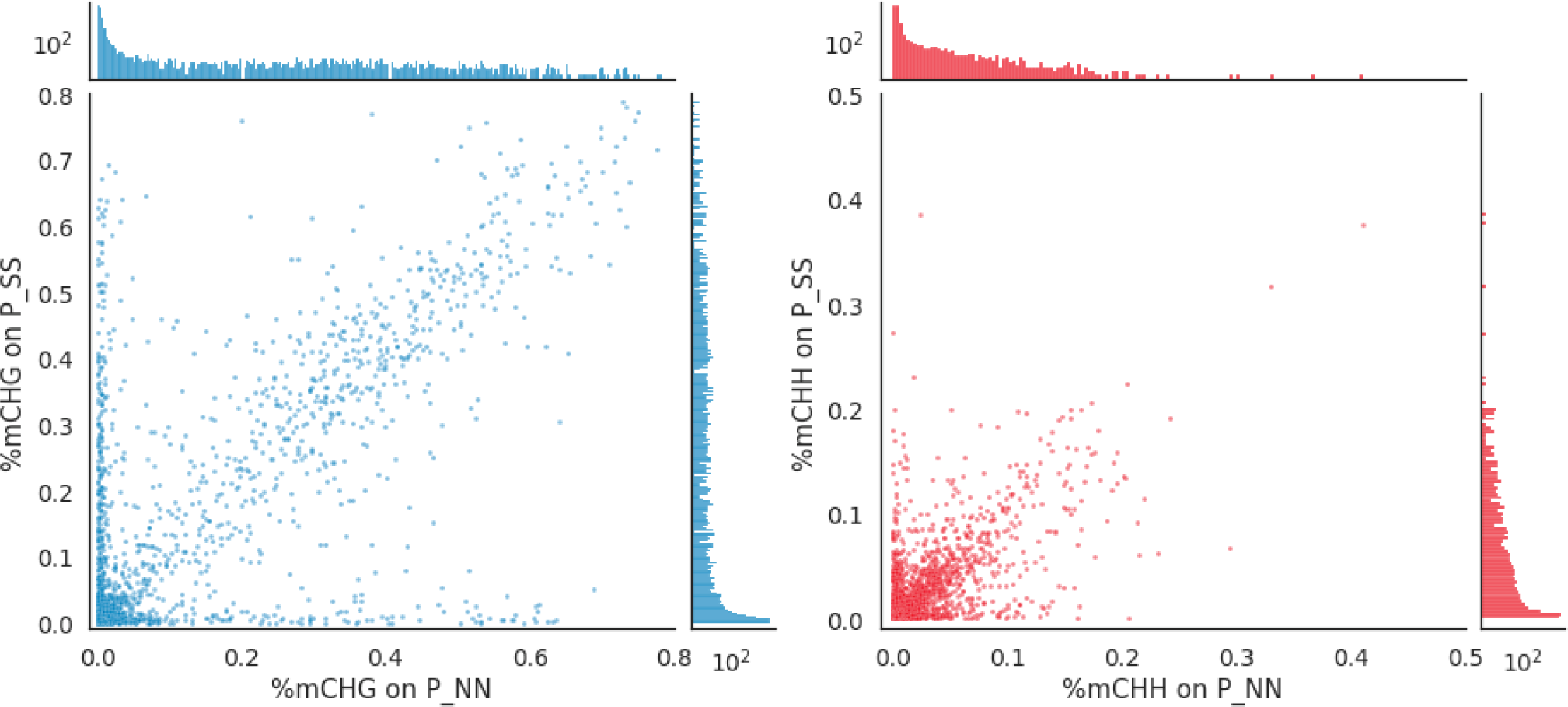

Supplement: S12 Fig — %mCHG and %mCHH on annotated protein coding genes (Araport 11) in parental lines N and S. We filtered out genes having any non-CG methylation on the gene-bodies to determine the gbM genes. (TIF) [file pgen.1010728.s018.tif]

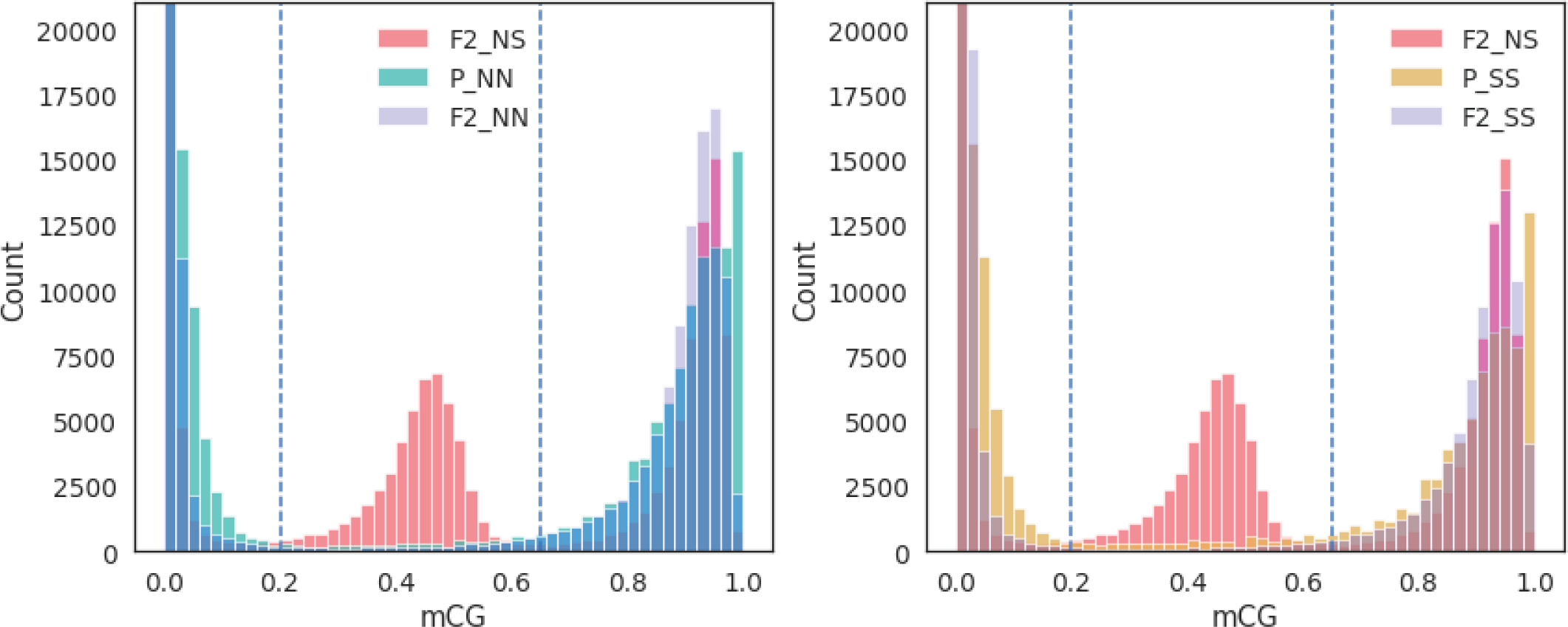

Supplement: S13 Fig — Histograms for methylation levels on gbM sites averaged across parental values (N in the left panel and S on the right panel), F2 individuals homozyogous for the same ancestry, and F2 individuals heterozygous for ancestry. There are far more sites with intermediate values in the parental than in the homozygous F2 data, although the former is also supposed to be homozygous. (TIF) [file pgen.1010728.s019.tif]

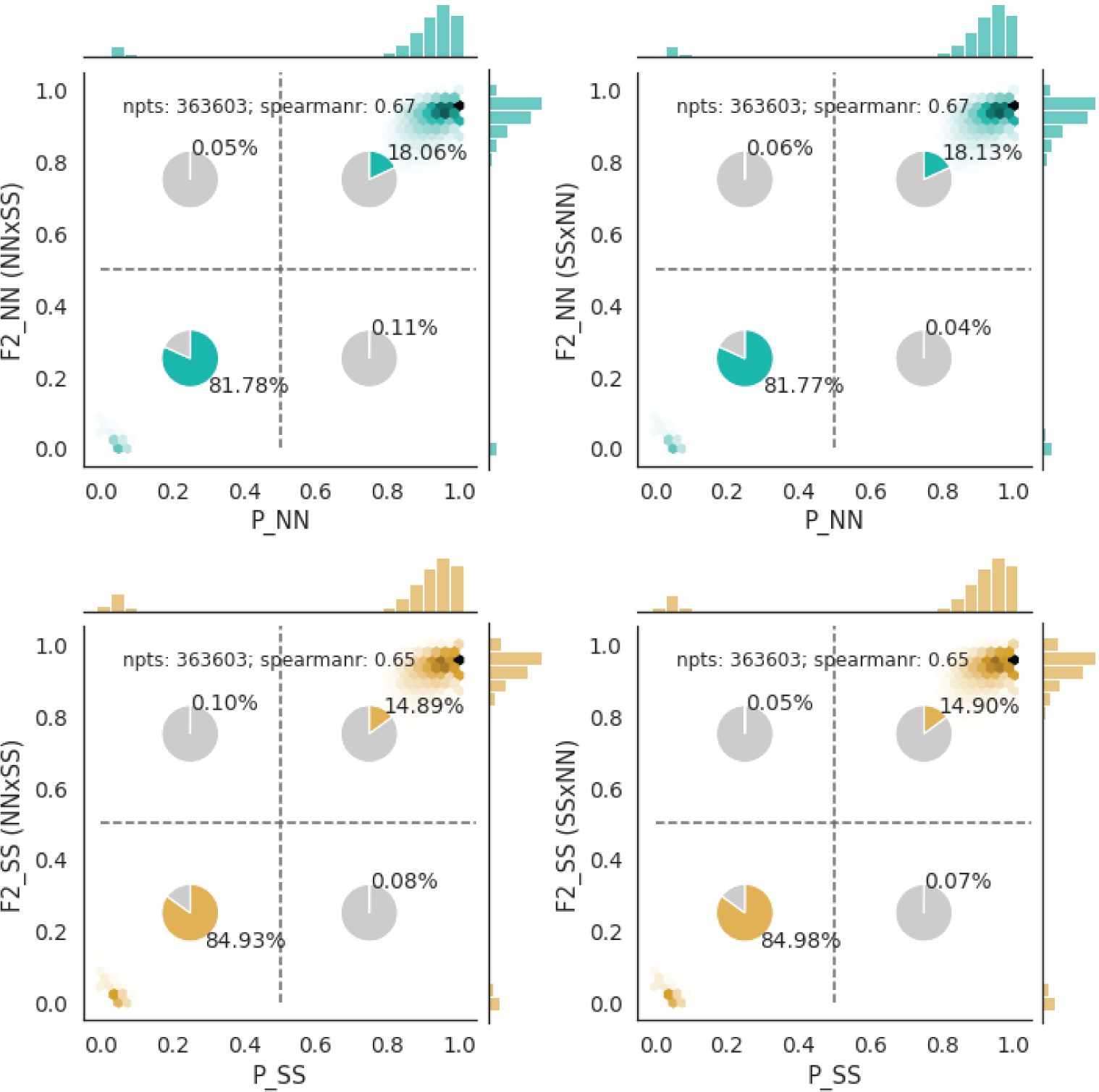

Supplement: S14 Fig — The plots compare average gbM for parents with average gbM for F2 individuals homozygous for the parental ancestry across sites, separately for the two cross-directions. Only data from chromosome 5 was used as all other chromosomes showed evidence of residual heterozygosity in the southern parental line (S1 Fig). (TIF) [file pgen.1010728.s020.tif]

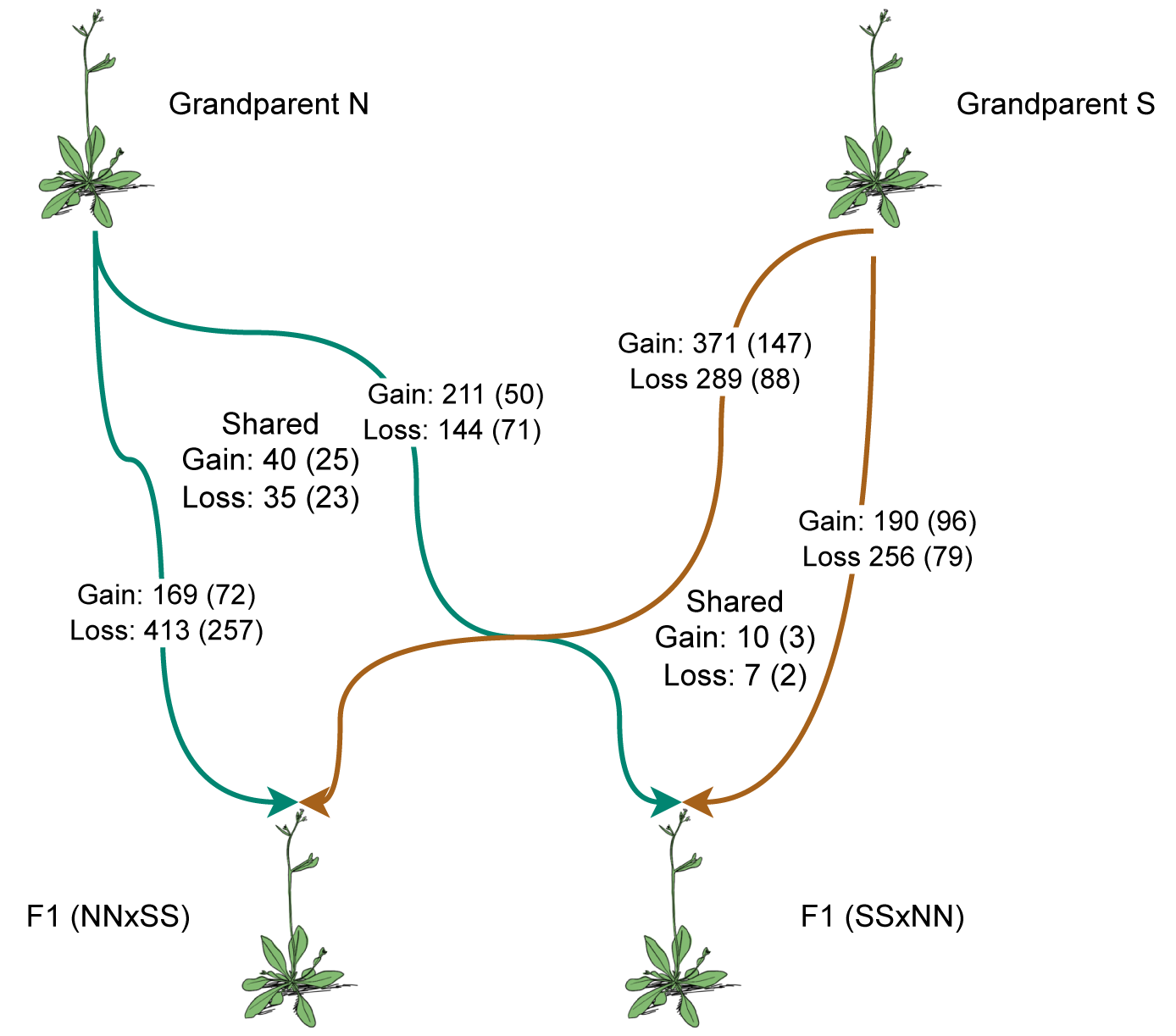

Supplement: S15 Fig — The transgenerational epimutation from S14 Fig are shown for each line-of-descent in the cross. “Shared” refers to the number of changed sites that are shared between the directions of the cross, separately for the northern and southern ancestry. The numbers in parentheses are for the sites that are differentially methylated sites between N and S (S16 Fig). (TIF) [file pgen.1010728.s021.tif]

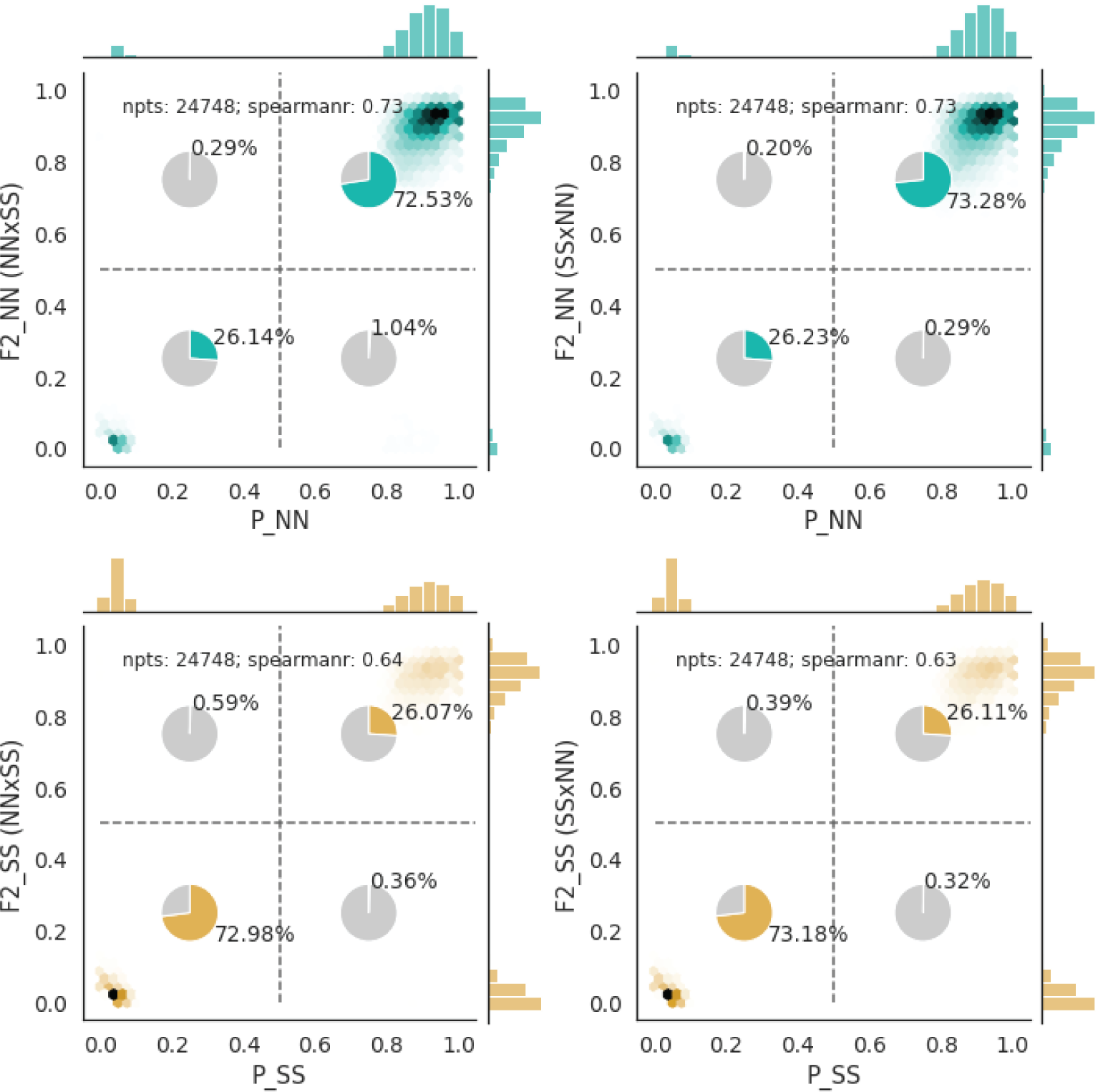

Supplement: S16 Fig — Same plots as S14 Fig but only sites that differ in methylation between the parental lines were used. (TIF) [file pgen.1010728.s022.tif]
