# Supplementary material for: On the causes of gene-body methylation variation in Arabidopsis thaliana
Source: PLoS Genet. 2023 May 4;19(5):e1010728. doi: 10.1371/journal.pgen.1010728 (PMC10187938; doi:10.1371/journal.pgen.1010728)
Supplement: Attachment — Submitted filename: Response to reviewers.pdf [file pgen.1010728.s023.pdf]

Response to Reviewers

Reviewer #1

This study details a unique genetic approach to try and identify genetic and environmental factors underlying variation in DNA methylation in the model plant Arabidopsis thaliana. Two distinct Swedish populations were used in a genetic cross due to their contrasting patterns and levels of gene body DNA methylation and transposon methylation. Whole genome bisulfite sequencing of hundreds of the resulting F2 progeny were used to test the impact of genetic and/or the environment.

This research showed convincingly that variation in methylation that originates in the populations is largely inherited in cis, as the variation observed mostly arose from loci that were distinct between the two original parents used in the cross and Mendelian segregation was observed. Changes in DNA methylation within gene bodies was almost exclusively limited to single symmetrical sites and the rates of loss was substantially higher that the rates of gains. Curiously gains were higher near pericentromeric regions of the genome. The rates of gains or losses didn't vary with temperature.

These variations were used in a linkage mapping approach to identify potentially causal cis versus trans genetic factors influencing this molecular trait. Significant trans-genetic associations for both gains and losses were observed, which is very exciting. Identifying the causal nature of these QTL will be important to further understand how variation in DNA methylation within gene bodies is shaped naturally between populations.

Overall, this is a nice study that takes advantage of a large cohort of F2 individuals to test and map variation in gbM between contrasting patterns in the parents. The comments below are intended to further improve this study.

We appreciate the positive feedback as well as the constructive comments.

1. In general I find Figure 3C difficult to interpret as presented.

2. It is reported that H2A.Z and variations in methylation are correlated. Please show a metagene plot of gains and losses of epimutations across gene bodies. H2A.Z is specifically localized to the 5' end of expressed genes, so if there is a direct effect the expectation would be that methylation variation is occurring at the same location.

We agree that this is difficult to interpret and possibly misleading. We have removed this panel from the figure and replaced it with a supplemental figure (S4) that shows that gains and losses are associated with different chromatin states as defined in Jamge et al. (2022). The original claim was based on the association between somatic deviation and a chromatin state associated with H2A.Z.

3. It is stated that no trans QTL are shared between gains/losses, but isn't there one on chr5?

Indeed. We have weakened the claim ("appear to be different") and now note that while the confidence intervals overlap, the peaks are far apart, and affect different loci. We cannot rule out that QTL are shared, but there is little reason to believe this.

4. How are sequencing errors, bisulfite conversion errors and other potential sources of errors impacting the interpretation of gains and losses given these are not directly modeled in this study?

Random errors are less of a problem given that most analysis rely on averages; bias is a different matter. We have added text to the relevant Methods sections discussing this.

5. I would consider expanding the discussion on the differences between loss and gain rates if space allows it.

We have considerably expanded the Discussion to deal with this and comments from the other reviewers.

6. Figure 6b would be better presented if it was converted to a density plot. It is difficult to know where most of the data points occur in these plots. Figure 7a should also be converted to a density plot.

Figure 6b becomes unreadable as a density plot because of the very uneven density of points along the x-axis. We feel the 90% intervals around the median (which were previously unexplained in the caption) convey the information better. Figure 7a has been converted as suggested.

7. I would recommend enlarging the text in most of the figures.

Agreed, we have gone through the figures to improve this.

Reviewer #2

The authors study CG gbM methylation changes in F2 populations obtained from a bi-parental cross between northern (NN) and southern Swedish (SS) accessions. They do reciprocal crosses to investigate parent of origin effects. The F2 individuals are obtained from seeds of a single F1(NS) individual. As a “control” they propagate the grand parental (NN or SS) lines by selfing for two generations (to match the generation of the F2 intercross). They use this design to try to approximate “epimutation rates” and compare them across N and S haplotypes. Also, using somatic deviations in CG methylation relative to the “ancestral state” as a molecular phenotype they perform QTL mapping in the F2 and identify genetic loci that contribute to somatic gains and losses of CG methylation, both in cis and in trans. One potentially interesting insight is that genetic variation leads to gbM variation via changing the epimutations rate.

The authors provide a substantial amount of new data, and it seems the bioinformatic / genetic analyses are generally carefully done. However, their results have alternative explanations that their current experimental design cannot rule out. Based on several pieces of evidence I think the authors are looking at a mixture of at least two processes that shape their results. First, a “classical” process of spontaneous epimutations as it has been studied in MA lines; and second, F1 methylome remodeling via TcM and TcdM events. My reading of their results indicate that their estimates of epimutation rates as well as their study of somatic deviations in the F2 are substantially influenced by the latter process If the latter processes contributes to their observations, their conclusions that genetic effects on epimutations rates shape natural variation in gbM in the long-term would be wrong as they F1 remodeling explanation is transient (see literature on F1 DNA methylation studies and paramutation). I think the authors could have addressed this alternative explanation with two additional modifications to their experimental design: 1. Take direct measurements on F1 hybrids (possibly even the one that produced seeds for the F2); and 2. generated more parental lines by selfing as controls, so that “classical” epimutational process could be directly contrasted within selected genomic regions (e.g. haplotypes under the cis QTL peaks). Without this, the interpretations/conclusions presented are questionable. I also think the authors could have done a better job explaining in more detail what they did. It is not straightforward following their analysis.

Many thanks for these comments, which we didn't understand until we googled "TcdM" and realized to our embarrassment that we had neglected some important papers that are obviously relevant as they dealt with methylation changes in F1s from crosses between A. thaliana lines, and even changes in the F2 generation (Greaves et al. 2012; Zhang et al. 2016; Greaves et al. 2016). After (re-)reading these papers, it is obvious that they should have been cited, and discussed. This has been rectified, and our paper is clearly better for it. We are grateful to the reviewer for having done a service to us and the readers. Before responding to the specific comments below, let us briefly respond to the statements above.

Most importantly, we absolutely agree that we are looking at a mixture of processes, but we feel that a dichotomy between a "'classical' process of spontaneous epimutation" and an "F1 methylome remodeling" is artificial. A. thaliana *is a sexual species, and the inbred lines we work with in the lab are ephemeral. Our starting point is that the differences between the lines we work with must reflect some combination of trans-acting genes and the environment, and our results show that both matter. The papers noted above also find evidence for trans-acting factors, but lack the power to map them. It is certainly not clear how persistent these changes are across generations, but we explicitly say this. The point is that, in a sexual organism, the observed epigenetic state must reflect both the action of trans-acting modifiers currently in the genome, and modifiers to which particular segments have been exposed in the past. Presumably some (environmentally dependent?) equilibrium is eventually reached, but we have no idea how long this takes. Thus, while we appreciate the suggestion for more F1 measurements, we would much prefer to trace the offspring many generations into the future. In particular we would like to know how probable it is that a somatic change becomes fixed in the next generation, and we note that estimating epimutation rates was a minor part of our paper.*

It is also notable that most of the changes seen in the paper above involved TEs, whereas we are looking only at gbM (and we filter out any genes with evidence of non-CG methylation).

We now cover these points, in particular in the Discussion.

Below are my comments:

- The section on estimating epimutation rates should clarify it if this is based on just gbM genes and CG context.

It is, and we have clarified this. In fact all our results are gbM only, and we eliminate genes with significant non-CG methylation. We also considered further eliminating genes with TE-methylation in introns, but the number is too small to affect our analyses.

- To estimate epimutation rates they used consensus calls in N parental replicates, and looked at consensus calls in NN homozygote F2, which would reflect the F1 state. Any difference they would see in the predicted F1 relative to the grand parental state (inferred from consensus parental calls) would be an epimutation. Any trans-generational epimutations that occurred during these two generations would give rise to differences between the inferred grand-parental and F1 states, and it should be possible to use this to estimate the epimutation rate. This strategy therefore also picks

up TcM and TcdM events that occurred in the F1 hybrids that are (partly) carried over to the F2. For paramutation this is well established. So, these hybrid events are factored into their estimates. There are several anomalies and observations that tell me that the later processes contribute substantially to their detected "epimutations".

We agree, and now clarify this in the Discussion as well as Materials and Methods. However, we note that "hybrid events" are not necessarily distinct from "epimutations".

1. Contrary to what the authors claim in the paper, their estimated rates do not look much like those for trans-generational epimutations detected with MA lines: they are much higher than the ones reported in the literature in studies of MA lines, the ratio of the rates is very different (TcdM events are most frequently observed); and – importantly- they have nearly opposite distributions across the genome compared to trans-generational epimutations (e.g. their gains peak in pericentromeric regions).

All valid points, and we have significantly expanded the Discussion to cover this. The one conclusion we should all be able to agree on is that further studies are required to determine whether changes that happen in relation to sex and outcrossing are irrelevant because they are only temporary, or whether changes seen in MA lines are irrelevant, because all the real action involves sex and outcrossing.

2. They observe parent of origin effects, which has also been reported to affect TcdM and TcM events.

We now note this.

3. They found evidence for "rapid changes in the F1 generation" during their filtering of sites, consistent with CG methylation changes originating from F1 methylome remodeling.

Sure, but, again, this is not categorically different from epimutations. There is an implicit notion here that these changes are temporary, and will return to some "natural" state — but what state will that be, in a rearranged genomes?

4. The overlap between epimutated sites between crosses is orders of magnitude higher than could be expected under any model of random epimutations (consistent with TcM and TcdM being largely systematic / reproducible processes).

Yes, we now note this.

5. Epimutation rates were higher for sites where the NN and SS parents were differentially methylated (TcM and TcdM events are proportionally more likely to occur in DMRs or SMPs; but I agree could be also due to the sites being more epimutable; we just don't know).

- While TcdM and TcM events in F1 hybrids are also relatively rare (based on the literature), they are still much more frequent than what would be expected under a spontaneous epimutation model (given the known rates), which could account for the

large rate differences relative to the literature. Finally, TcM events (but also TcdM events) are sRNA mediated. While a strict definition of gbM genes will unlikely have sRNA targets, I have noticed that the authors work with a list of >10K classified gbM genes (a strict list would be around 5K). Their liberal list likely includes genes with some non-CG methylation (perhaps in their introns), which likely harbor sRNA targets (even if this is just a fraction of all genes). The fact that most of their gain and loss events occur in pericentromeric regions would be further consistent with a Tcdm and TcM process as being the major driver behind these epimutation rates they get (note that the gain rate and loss rate are not highest in pericentromeric regions for trans-generations spontaneous epimutations). The sRNA argument could be bested using known sRNA target sites or by direct measurements.

The TcM and TcdM events discussed in the papers above are indeed overwhelmingly siRNA-dependent, but they are also overwhelmingly affecting TEs, which we don't consider in this paper. As noted above, we eliminated all genes with evidence of non-CG methylation.

However, we note that the papers above also found evidence for changes not associated with siRNA, and we absolutely agree that what we see is associated with the TcM and TcdM phenomena. We do not agree that this is unrelated to epimutations (in nature, not in MA lines) — we simply do not know.

Finally, note also that while our gains were indeed associated with pericentromeric regions, losses were not. It is also our understanding that the estimated rate of trans-generational gains on genes are higher in pericentromeric regions (Denkena, Johannes, and Colomé-Tatché 2021).

- I think the possibility that we are dealing with many Tcdm or TcM induced methylation changes in the F1 potentially affects their conclusions about somatic deviations. There are at least two considerations: First, although the F2s come from a single F1, the F1 gametes constitute a population of different haplotypes. TcdM and TcM events can continue in the germline, or – more likely – be partly "corrected" in the germline. The correction is likely stochastic as permutation in A. thaliana has shown that F2 and F3 individuals only partly maintain F1 induced methylation changes (some have it while others don't). The "correction" of such F1 events could also continue during F2 development, thus leading to somatic deviations. That these deviations are mappable could then relate to the molecular pathways that are involved in the correction process. The fact that many non-CG genes are under the detected QTL peaks would suggest to me a possible involvement of RdDM. Note that this could also explain the detected trans-effects as those trans effects are also potentially active in the gametes. Again, I think all of this could be tested with sRNA data or – as I mentioned above – with more data at F1 and parental level. Such data would have allowed the authors to delineate how much of the CG methylation changes are just a byproduct of having passed through an intra-specific F1 hybrid.

Good points, but we note that "corrected" is in quotes for good reason — what is correct here? Why would the somatic changes be reverting to some Platonic ideal? On the contrary, with respect to growth temperature, the somatic deviations point in the same direction as the somatic deviations observed in the parental lines (Fig. S8), consistent with none of these genotypes being at equilibrium in its current environment. Our suspicion is that the observed epigenetic state will gradually converge to some equilibrium determined by genotype and environment. We 100% agree that more studies are needed, but rather than more F1 data, we would like to see F3, F4, etc.

We have expanded Discussion to emphasize this.

- In their analysis of somatic deviations it should be clarified if the analysis is restricted only to CG gbM methylation If so, does the window approach and the plotting of gain/losses normalized by gbm density?

All analyses are restricted to CG gbM, and the plots are per-site, and are thus independent of gbM density. We have also mentioned this in the Methods section.

- The authors use a binomial model to test the hypotheses that CG methylation changes occur in a site- and individual independent manner. For gains they cannot reject this model, while for losses they can. For gains, how can this be reconciled with their other finding that gain CG "epimutations" are autocorrelated and – by extension – with the model proposed by Brifa et al (2022, BioRxiv)? These two results seem to be contradictory. The strong binomial rejection for losses is consistent – again – with a systematic TcdM correct process (TcdM events are more frequent in many of the F1 methylation studies).

This is a misunderstanding. The autocorrelation shown in Fig. 6 and in Brifa et al. (2022) refers to non-independence between sites along the genome. The non-independence rejected by the binomial model is between individuals. There is thus no contradiction. We have added a sentence to avoid confusion.

- That trans-generational epimutations occur in clusters (or as DMRs) has been shown by Denkena et al. (2021, Heredity). This study should be cited.

We agree, thanks!

- In the QTL analysis, it is unclear if the 500 kb windows only focused on gbM genes.

Everything is. We have clarified this.

- While there are lots of reasons why the GWAS could not replicate the F2 QTL, one interpretation is – again - that process that leads to long-term stable methylation differences between accessions (here NN and SS lines) in nature is different from the one that pick up for somatic deviations in their F2 mapping.

Sure, but the GWAS was done on somatic deviations.

- For the cis QTL it would have been useful to have data on multiple parental lines at G2. This would have yielded direct comparisons of the epimutation accumulation in selfed lines on haplotypes under the cis QTL peaks and those haplotypes when passing through the F1 and/or the impact of trans-effects.

We agree it would be really useful to test if one could directly compare parental lines with haplotypes in F2. We would definitely need multi-generational data to test this.

- The authors write: ". It seems more likely that the effects are a consequence of the epigenetic differences that we know exist. As mentioned previously, deviations are associated with the underlying chromatin state (Fig 3), suggesting the local epigenetic state influence them. "

Where do the authors think the chromatin state differences are coming from? If not genetic?

The environment? Or both in interaction? This is stated in the Introduction. We now return to it in the Discussion.

- How many CG sites are expected to be found in 30bp? How does this result relate to the autocorrelation epimutation model proposed by Brifa et al (2022, BioRxiv)?

It varies, but not many. We believe our results are entirely consistent with those of Brifa et al (2022) and mention this is the discussion.

Line 12: mCHG → mCG

Done.

Line 19 and 22: Quantify the range of gbM (e.g. from as low as to as high as….). This would put things into perspective. One can map QTL for phenotypes that vary very little, provided the variance is low enough. The differences are mentioned in the methods. Should be brought into the intro, for my taste.

Those results are also in the cited papers, but the range will depend on which genes are included. For what it's worth, the full range in the 1001 Genomes date the way we calculate it is 7% to 18.5%, with a mean of 13%. We do give precise numbers for the two parental lines we are working on in this paper (Fig. 1 and discussion thereof): we're considering over 650k exonic CG sites, and roughly 25% of these are methylated in at least one parental line; 45% of these differ between the parents, and, of these, 70% are only methylated in the northern line. The differences are substantial.

Lines 37-39: This does not follow from this argument. For example, being fixed for slightly deleterious alleles in DNA methyltransferases will lead to bias epimutation ratio, shift steady state methylation (lead to mean differences) and should therefore be mappable. In selfing species you are sitting in a "fixed" genetic background for long enough.

Yes, in a selfer the direct “phenotypic” effect of a modifier will, on average, be 1/(1-s) higher, and this helps, but does not change the fundamental validity of the argument. Depending on how stable CG mutation is (and it seems to be rather stable), most of the variation will still reflect history, not genetics. We modified the text to clarify this.

Lines 44-47: Clarify sentence...can map it despite stability requires a slightly more explanation why this should be so.

Agreed, clarified.

Line 79: “massive”; reword

Replaced with “roughly 75-fold”.

Line 162: a couple of percent; be more precise.

We could certainly give two numbers, but that would in our opinion not actually be more precise, and arguably misleading. For the losses we say “about 5%”. All these estimates are highly model-dependent, and the absolute number doesn’t matter to our argument. All we want to get across is that the effects are small, but not trivial, and that the two gain QTL appear to have smaller effect than the two loss QTL: writing “a couple of percent” vs “about 5%” accomplishes this.

Reviewer #3

In their study, Pisupati and colleagues investigate what determines the variation of gene-body DNA methylation, which has been known to differ between natural accessions of A. thaliana. They use an F2 population derived from a cross of a northern and a southern Swedish genotype with high and low gbM, respectively. The authors find that somatic deviations occur mostly at sites that differ in terms of DNA methylation between the parents, and that gains and losses of methylation follow very different patterns. In a QTL mapping approach, the authors identify different loci associated with either gains or losses, most in cis but some in trans, although they cannot identify, let along validate, candidates.

The study takes an interesting angle at the question of what causes DNA methylation variation and at which rate this kind of variation arises. It aligns well with several recent studies, mostly by the Gaut, Schmitz and Johannes labs. My biggest criticism is therefore that the authors missed the opportunity to contrast their results in more detail to the findings from some of these studies.

We agree that we should have done better relating our results to other studies, not only the ones noted here, but also the ones alluded to by the other reviewers. We have significantly expanded the Discussion to amend this.

Apart from that, the manuscript is clear and concise and nicely complements the existing body of literature on the topic. I have listed below some points where information was missing or where statements were unclear.

Minor comments:

- Unless I missed it, it seems that the authors did not provide a summary of sequencing stats. It would be informative to see the per-sample average coverage and, more importantly, the bisulfite conversion rates. Differences in conversion rates could potentially affect methylation calls. Given the number of individual samples, I doubt that the results would be seriously affected, but supplying these data should be part of the standard material supplied with this kind of study.

We have added a supplementary figure (S10) and explained how we incorporate bisulfite conversion rates when estimating somatic deviations in the Methods section.

- The title: Do the authors really provide insights into the “cause” of DNA methylation variation? The manuscript does not even speculate on potential candidate genes in the QTL mapping intervals that could explain the variation, and it surely does not provide an explanation. I therefore find the title somewhat misleading.

Well, we demonstrate that the somatic deviations have genetic, epigenetic, and environmental causes. We argue that these somatic deviations results in trans-generational changes, and thus that the geographic patterns observed in nature might reflect this kind of G x E. In this sense our paper is very much about the causes of gbM variation! We now make this clearer in the Discussion.

- Figure 2 left me a bit mystified. I think it would be helpful to explain the symbols, colors, etc. in the legend. Particularly in the lower panel, the numbers and the varying number of rows of “methylation calls” are not self-explanatory.

We have added a legend.

- Line 136: effecting -> affecting

Done.

- Line 155: “the 500 kb size was empirically determined” -> How? If I am not mistaken, this is mentioned nowhere in the text.

Thank you for noticing. We chose 500 kb as it resulted in approximately 10k cytosines per window, a magnitude needed given the low rate of events (around 10^-4). We have explained this in Methods.

- Fig S1: what is the purpose of the “uncrossed” and seemingly un-sequenced individuals in the 2nd row from the top (outer left and outer right)?

None. We have removed them.

- Fig S3: this may be due to the way the data is presented here, but at least in these graphs, the differences between the genotypes seem drastic. From the legend, it appears that this figure shows ALL mCG sites, not only the gbM ones. Is that the case? If so, it would appear that SS is almost completely depleted of overall CG methylation!?

The figure shows only differentially methylated sites, and the SS values are low because relatively few sites are methylated in this line only. We have clarified this in the figure caption.

- Fig 3D: “DMC” is not explained anywhere, nor does it appear anywhere in the text.

Thanks; fixed.

References

Denkena, Johanna, Frank Johannes, and Maria Colomé-Tatché. 2021. “Region-Level Epimutation Rates in Arabidopsis Thaliana.” *Heredity* 127 (2): 190–202.

Greaves, Ian K., Steven R. Eichten, Michael Groszmann, Aihua Wang, Hua Ying, W. James Peacock, and Elizabeth S. Dennis. 2016. “Twenty-Four-Nucleotide siRNAs Produce Heritable Trans-Chromosomal Methylation in F1 Arabidopsis Hybrids.” *Proceedings of*

the National Academy of Sciences of the United States of America 113 (44): E6895–6902.
Greaves, Ian K., Michael Groszmann, Hua Ying, Jennifer M. Taylor, W. James Peacock, and Elizabeth S. Dennis. 2012. “Trans Chromosomal Methylation in Arabidopsis Hybrids.” *Proceedings of the National Academy of Sciences*. http://eutils.ncbi.nlm.nih.gov/entrez/eutils/elink.fcgi?dbfrom=pubmed&id=22331882&retmode=ref&cmd=prlinks.
Jamge, Bhagyshree, Zdravko J. Lorković, Elin Axelsson, Ramesh Yelagandula, Svetlana Akimcheva, and Frédéric Berger. 2022. “Transcriptional Activity Is Shaped by the Chromatin Landscapes in Arabidopsis.” *bioRxiv*. https://doi.org/10.1101/2022.06.02.494419.
Kakoulidou, Ioanna, Robert Stefan Piecyk, Rhonda C. Meyer, Markus Kuhlmann, Thomas Altmann, Caroline Gutjahr, and Frank Johannes. 2022. “Parental Pericentromeric Methylation Status Drives Methylome Remodelling and Heterosis in Epigenetic Hybrids.” *bioRxiv*. https://doi.org/10.1101/2022.09.29.510107.
Muyle, Aline M., Danelle K. Seymour, Yuanda Lv, Bruno Huettel, and Brandon S. Gaut. 2022. “Gene Body Methylation in Plants: Mechanisms, Functions, and Important Implications for Understanding Evolutionary Processes.” *Genome Biology and Evolution* 14 (4). https://doi.org/10.1093/gbe/evac038.
Muyle, Aline, Jeffrey Ross-Ibarra, Danelle K. Seymour, and Brandon S. Gaut. 2021. “Gene Body Methylation Is under Selection in Arabidopsis Thaliana.” *Genetics* 218 (2). https://doi.org/10.1093/genetics/iyab061.
Yao, Nan, Robert J. Schmitz, and Frank Johannes. 2021. “Epimutations Define a Fast-Ticking Molecular Clock in Plants.” *Trends in Genetics: TIG*, May. https://doi.org/10.1016/j.tig.2021.04.010.
Zhang, Qingzhu, Dong Wang, Zhaobo Lang, Li He, Lan Yang, Liang Zeng, Yanqiang Li, et al. 2016. “Methylation Interactions in Arabidopsis Hybrids Require RNA-Directed DNA Methylation and Are Influenced by Genetic Variation.” *Proceedings of the National Academy of Sciences of the United States of America* 113 (29): E4248–56.
